# Development of a generic decision guide for patients in oncology: a qualitative interview study

**DOI:** 10.1186/s12911-025-02960-6

**Published:** 2025-03-10

**Authors:** Lia Schilling, Jana Kaden, Isabel Bán, Birte Berger-Höger, on behalf of the TARGET group

**Affiliations:** Institute for Public Health and Nursing Research, University of Bremen, Bremen, Germany

**Keywords:** Shared decision-making, Decision support interventions, Oncology, Patient participation, Informed choice, Guidance

## Abstract

**Background:**

Many patients with cancer want to be involved in healthcare decisions. For adequate participation, awareness of one’s own desires and preferences and sufficient knowledge about medical measures are indispensable. In order to support patient participation, a decision guide for patients with cancer was developed as part of a larger project called TARGET, which specifically aims to improve the care of patients with rare cancer.

**Methods:**

The development of the decision guide took place from 08.2022 to 03.2023. The decision guide is a single component of a complex intervention that aims to facilitate decision support in cancer care for patients. For the development, existing development and evaluation studies of Question Prompt Lists (QPLs) were identified through systematic literature searches in the MEDLINE via PubMed, PsycInfo, and CINAHL databases. The decision guide was pre-tested for feasibility, usability, completeness and acceptance with the target groups through guided individual interviews. Sociodemographic data were collected anonymously. An expert review was conducted. The verbatim transcribed interviews were analysed using content analysis according to Kuckartz with MAXQDA. The guide has been iteratively optimized based on the results.

**Results:**

A generic decision guide for patients with cancer for diagnostic or treatment decisions was developed in both PDF web-based formats, based on the Ottawa Personal Decision Guide. It was supplemented with decision-related questions from QPLs for patients with cancer. The pre-test comprised seven expert reviews of (psych)oncologists and experts in evidence-based health information and ten interviews with cancer patients (*n* = 7), family relatives (*n* = 2), and one caregiver. The results were coded into nine main categories. The results indicated a good feasibility, usability and acceptability of the guide. The tool was perceived as comprehensive and appropriate. Individual elements were identified as modifiable for better comprehensibility. The target audience appreciated the decision guide as a good support option.

**Conclusion:**

The decision guide is potentially a useful support option for patients with cancer facing medical decisions in their further course of treatment. In the TARGET project, it will be made available to patients and can be supplemented with decision coaching. Further steps for implementation into healthcare structures are necessary.

**Clinical trial number:**

Not applicable.

**Supplementary Information:**

The online version contains supplementary material available at 10.1186/s12911-025-02960-6.

## Background

The inclusion of patients in decisions about their health is an explicit goal of the German National Cancer Plan [1]. Many cancer patients themselves want to participate in this process [2, 3]. The promotion of active patient participation in the decision-making process can be effectively facilitated through the implementation of shared decision-making (SDM) [4, 5]. SDM and the provision of evidence-based decision aids can lead to more informed decisions, enhance patient knowledge and reduce decisional conflict [6–9]. By using decision aids, patients can also be clearer about their priorities [7]. However, due to perceived knowledge gaps and existing role models, patients are often not sufficiently prepared to participate in decisions about their health [10–12].

SDM is an interactive process that includes all relevant parties (health care team, the patients and their relatives) in the decision-making process, fostering the comprehensive exchange of all decision-relevant information. A pivot point of this approach is the provision of information on all available medical options in an understandable and non-directive way by the healthcare team as well as the evaluation of this information by patients based on their needs and preferences [13, 14].

Decision-making in oncology can be highly complex since treatment options have different risk-benefit profiles. In order to decide, patients need to be able to process complex information in an emotionally demanding situation. This requires well-informed and educated patients [15, 16]. To facilitate informed, values-based decision-making and SDM various decision support interventions exist [17]. These include evidence-based health information, decision coaching, patient decision aids and guidance to support decision-making. Guidance provides patients with structured support to facilitate a self-directed approach to the process of making informed health decisions. Guidance can be used by patients independently or in the context of a professional consultation, for example with the support of decision coaches. Guidance can include step-by-step approaches for making a decision, a worksheet for patients to clarify their values regarding the different options that can be shared with their healthcare team, a lists of questions which patients would like to ask their clinicians, or automated summaries of patients’ priorities and needs. By helping patients clarify their values in relation to the potential benefits and risks of different options, guidance promotes confidence and understanding in health decision-making [7, 18, 19]. The International Patient Decision Aid Standards (IPDAS) collaboration has identified guidance as a critical element in supporting self-directed decision making [18, 20]. It can be included into patient decision aids or serve as a complementary resource [19]. A decision guide is therefore a structured tool designed to support patients in their decision-making process. It can include various elements to help patients understand their values and preferences regarding all available options and to be well prepared for their decision-making process.

Decision aids are evidence-based tools designed to help patients make informed choices about their health cate options [7]. They can complement clinicians’ counselling by explicitly stating the decision to be made and providing evidence-based information about health conditions, available options, benefits, harms, probabilities and uncertainties. When using decision aids compared to standard treatment, patients are significantly more aware of their available options and have a better understanding of risk. The decisions are more often in line with the patient’s own values and the decisional conflict decreases [6, 7, 21]. There are only a few, freely accessible decision aids for patients in oncology available in Germany. This can be explained by the complex decisions to be made in oncology and the time-consuming process of the development and tailoring to specific target groups [22]. Especially the rapidly changing evidence and the differentiation of treatment with regard to personalized targeted therapy concepts complicate the development of decision aids. Furthermore, the complexity increases due to the variety of rare cancer types and their treatments, making the provision of decision aids for all these conditions currently challenging [16, 23].

Therefore, our aim was to develop and pre-test a generic decision guide for patients with cancer to support their participation in decision-making processes. We aimed to support patients to identify and address their decisional needs when facing decisions in oncology.

## Methods

We conducted a qualitative interview study to pre-test a a single component of a complex intervention developed in accordance with the UK Medical Research Council’s (MRC) Framework for the Development and Evaluation of Complex Interventions [24]. The single component that we developed and pre-tested is a generic decision guide. The results are reported in accordance with the Criteria for Reporting the Development and Evaluation of Complex Interventions in healthcare (CReDECI 2) (Additional File 1) [25] and the Consolidated criteria for Reporting Qualitative research Checklist (COREQ) (Additional File 2) [26].

This study is part of a larger project called TARGET (the Trans-sectoral Personalized Care Concept for Patients with Rare Cancers). TARGET aims to improve the care of patients with rare cancer diseases in the model region of southern Bavaria and focuses on strengthening the network between service providers, both between sectors (outpatient and inpatient) and within sectors (e.g. between different medical disciplines) through the use of telemedicine [27]. By optimizing transsectoral cooperation and coordination of care, it seeks to enhance patient involvement and improve the medical care coordination. SDM should be promoted through decision support interventions (decision guidance, decision coaching and training of healthcare professionals in SDM). This study reports on the development and pre-testing of the generic decision guide for patient in oncology which can be used alone or combined with decision coaching. Due to its generic nature, the guide is not limited to rare cancers, but can also be used for non-rare cancers.

### Development

In order to identify potentially relevant questions and information needs of patients with cancer, we conducted a systematic literature search. MEDLINE, PubMed, PsycINFO and CINAHL were searched for development and evaluation studies of QPLs from 23.06.1973–15.08.2022. QPLs are structured lists of potentially relevant questions that patients in oncology can ask their doctors [28–30]. QPLs have been shown to help patients ask more questions during consultations [31] and increase the probability that they will seek information and express preferences about their care [32]. Our core search consisted of the term’s oncology, cancer, tumor, malignan* or neoplasms combined with terms related to question prompt lists (Additional File 3). The screening of the titles and abstracts was carried out independently by two researchers (BBH and LS) independently via the screening tool Rayyan. Disagreement between the reviewers was resolved by discussion. We included studies with available QPLs for patients in oncology. The focus of the QPLs was on potentially relevant questions. This refers to questions that enable patients to ask and find out everything they need to make a decision: their condition, all available options, their advantages and disadvantages, possible side effects and the implementation of their decision (e.g. “How likely is it that the treatment will control my cancer?” or “What are the pros and cons of each treatment option?”). QPLs that are specific to a cancer entity, treatment or for surgery were excluded. Studies that did not report original data on QPLs were excluded. Both, English and German studies were included, without any time restrictions. Systematic reviews were excluded, but the reference lists were checked for studies that fulfil the inclusion criteria.

To cluster the questions, we initially reviewed the included lists of question prompts and inductively derived main categories. In a second step, the remaining questions were then assigned to the main themes deductively. Duplicate questions were removed. The final list of identified questions from the QPLs was integrated into the decision guide as a separate chapter. We used the Ottawa Personal Decision Guide (OPDG) [33] as basis for our guide. The OPDG has proven to support in the decision-making process and was designed to assist individuals confronted with challenging decisions that will impact their health or social lives [33]. The OPDG is highly versatile and suitable for a wide range of decision-making situations [33]. Therefore, it was well applicable for our aim to develop a generic decision guide for decisions in oncology. The OPDG is based on the Ottawa Decision Support Framework (ODSF) which can be used to develop and evaluate decision support tools. The ODSF is an evidence-based framework that has been developed to assist individuals in navigating health and social decisions. The framework proposes that optimal decisions are achieved when the individual decisional needs are identified, and appropriate decision support is provided [17].

### Pre-test

To test feasibility, usability, comprehensiveness and acceptance semi-structured guided individual interviews with the target group were conducted. To ensure the accuracy of the content and for the implementation in clinical practice, (psych)oncologists and experts in evidence-based health information reviewed the decision guide and provided feedback. The experts were part of the TARGET team.

#### Setting and sample

We included cancer patients aged 18 or older and who had a cancer diagnosis within the last five years or relatives of cancer patients who have accompanied a person with cancer within the last five years. Patient with rare and non-rare types of cancer were included. To recruit participants, a flyer about the study was disseminated through cancer support groups, German Cancer Society websites and mailing lists and also via the researcher’s network. Interested people were asked to contact the research team to get more information about the study.

#### Data collection and analysis

Due to the vulnerable target group, we decided to use semi-structured, guided individual interviews. This approach can address the specific needs of each participant and provide a safe environment where they could share their personal story. Participants’ needs can be addressed individually and they have the opportunity to take a break or end the interview at any time.

The interviews were conducted online via the video conference platform ZOOM or on-site by two researchers (JK, LS) with a public health (LS, JK) and nursing background (JK) and experience in qualitative research (JK). The participants received the guide at least one week before the interview, either digitally or by mail, depending on their preference. The interviews were audio recorded with an external recorder and transcribed verbatim. The transcripts were not returned to the participants for checking. Sociodemographic data of the participants were collected anonymously via a questionnaire. These included information on age, gender, level of education and cancer history. Written informed consent was obtained from all participants prior to the interviews. If participants made notes in or about the decision guide, they were ask to sent these to the research team after the interview.

The interview guide (Additional file 4) was divided into the following main themes: First impression, User-friendliness/Usability, Comprehensibility/Clarity, Completeness, Acceptance and Graphical presentation. The participants were asked to give their assessment against the background of their personal experiences with the disease and related decisions.

The qualitative content analysis was carried out by two researchers (LS and IB) according to Kuckartz [34] with the software MAXQDA. A predefined category set based on the interview guide was applied to the interview data and supplemented by inductively derived subcategories during coding. The final category system (Additional File 5) was applied to the entire material in a final analysis loop. The analysis was conducted independently by IB and LS. Both have fully coded all interviews. After that, both researchers reviewed the entire coded text material collaboratively, agreeing on code selection and code length. Based on the results, the decision guide was iteratively optimized.

## Results

### Development

**Fig. 1:**
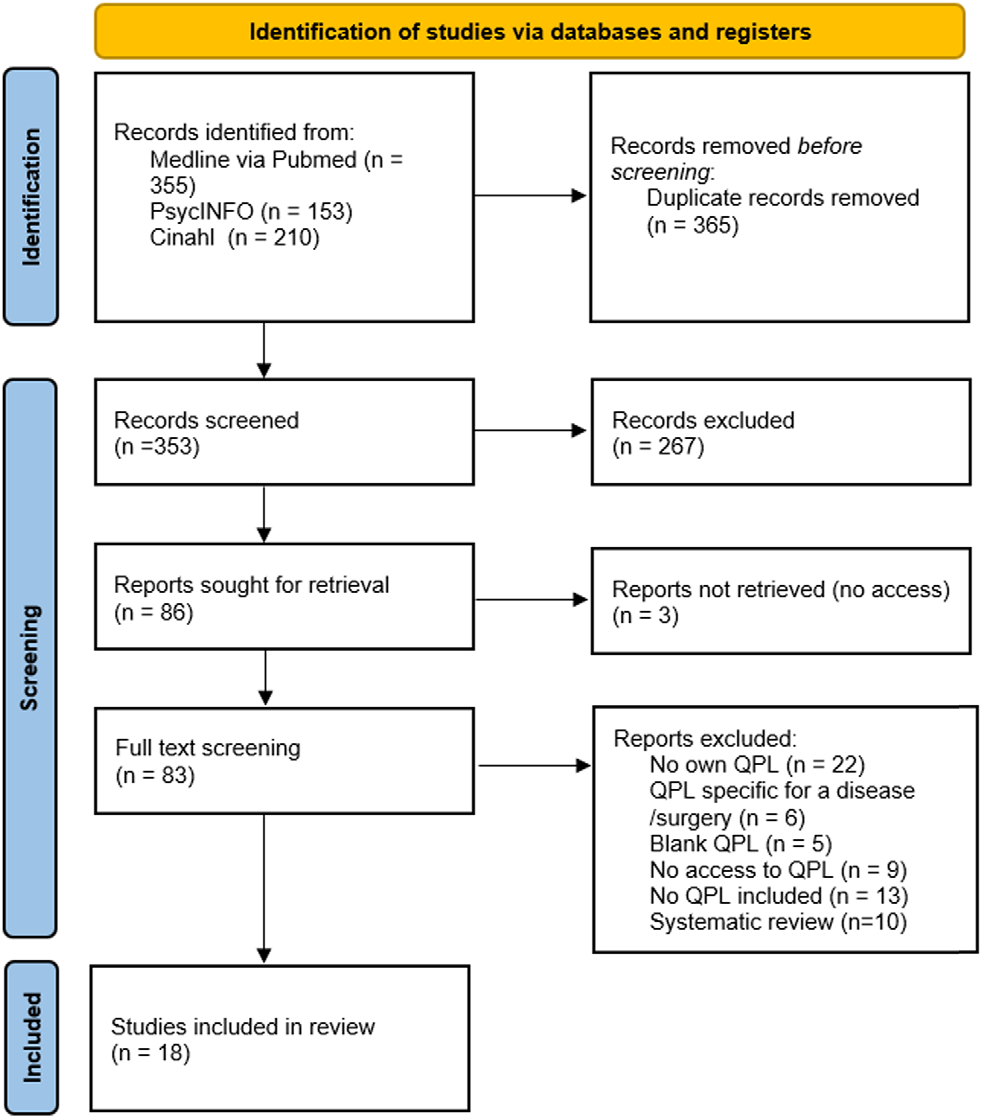
PRISMA flow chart

In our systematic research for existing decision support tools, we identified QPLs as a useful component for our decision guide. After our screening process, we identified 18 potential studies reporting on QPLs [28, 30, 31, 35–49] (Fig. 1).

From the 18 studies, a total of 596 questions emerged. The number of questions in the QPLs varied from 11 to 110 questions. We clustered these questions into seven main categories: diagnostics, prognosis, treatment options, symptom and side-effect management, clinical trials, support and palliative care. Duplicate questions and non-generic questions, such as questions about specific treatments, diagnostic tests or medications, were not included. This resulted in 61 questions for our decision guide.

Based on the OPDG [33], we developed a German generic decision guide for diagnostic and treatment decisions in oncology which is available in an editable and printable PDF format (Additional File 6) and in a web-based version through LimeSurvey [50]. The guide is designed to empower patients to ask for essential information about their available options and to assess their preferences in the context of these choices. Additionally, it is also intended to provide guidance for dealing with specific decisional needs.

### Structure and content of the decision guide

The decision guide comprises 19 pages and is structured to support patients in navigating through their decision-making process and to document their preferences. We used a large font size for better clarity and the guide includes headings and subheadings that act as a navigation tool, allowing patients to identify and focus on the sections most relevant to their individual needs. In addition, there is plenty of free space on the pages for the patient to take notes. The guide is structured into three main sections: “My questions” (two pages), “My options” (eight pages), and “What am I missing?” (six pages). The introduction and imprint comprise three pages. Based on the introduction and brief instructions, patients can decide which parts of the guide are particularly relevant to them. In the first section, patients should document their current status with regard to their upcoming decision. At the beginning, patients are asked to assign their decision to one of the decision types (diagnostic or treatment). We included a section called “Where do I stand?” to help people get more information about their condition and prognosis.

The second part allows patients to list and evaluate the different options in a table. Patients can use the table to write down their options, noting the perceived advantages and disadvantages of each, and to rate these factors. The first option is predefined as either “no further diagnostics” or “none of the proposed treatments” depending on the type of decision. This should help patients understand the potential benefits and harms of each option in comparison to “no further diagnostics” or “none of the proposed treatments”. It also encourages consideration of these as reasonable options, particularly in palliative care situations where best supportive care may be preferable to active treatment. This aligns with the criteria set by International Patient Decision Aids Standards (IPDAS) and the NICE Guideline for Shared Decision Making [18, 51]. In addition, to be transparent about the medically reasonable options, the guide includes a field where patients can fill in which option(s) the medical expert team (tumor board) recommends.

The 61 decision-relevant questions extracted from the QPLs were integrated into this part and are placed after the table of options. The 61 decision-relevant questions extracted from the QPLs were integrated into this part and are placed after the table of options. The questions are grouped by topic so that patients can recognise which questions may be relevant to their decision. In preparation of consultations with their treatment team, patients can mark and rank order personally relevant questions that should be clarified with their treatment team. In the LimeSurvey version, it is possible to drag and drop the questions in order to prioritize them.

In the last section, patients can identify and reflect on their decisional needs such as information deficits, decision uncertainty and inadequate support with the help of various questions and the SURE test [52]. The SURE test is an instrument to screen for decisional conflict using four brief questions [52, 53]. In addition, patients are assisted to reflect on potential support opportunities. Finally, patients can document how they will proceed with their decision and their next steps. In the LimeSurvey version, it is possible to create and print a summary of the completed guide.

## Results of the pre-test

### Participants

A pre-test was conducted from November 2022 to March 2023 involving ten participants, with seven of them being female (Table 1). A potential participant had to withdraw their verbal consent due to their health condition. The median age of the group was 58.5 years. Participants included seven persons affected by cancer, two family relatives and one healthcare professional specialised in the care of people with disabilities who was caring for a cancer patient. The median time since diagnosis for participants diagnosed with cancer was 4 years. Three patients were still undergoing treatment at the time of the interviews.

**Table 1:** Baseline characteristics

Sex (*n* = 10)	Female	7
	Male	3
Age in years; median (range) (*n* = 10)		58,5 [27–69]
Education (*n* = 10)	Secondary School Certificate, Intermediate School Certificate (also transition to 11th grade of High School/Comprehensive School)	1
	University of Applied Sciences Entrance Qualification, High School Diploma, Vocational Diploma, Vocational Qualification (Vocational School)	1
	High School Diploma and Vocational Diploma (Vocational School)	1
	Completion of a Technical School, Vocational Academy, Technical Academy, University of Applied Sciences, University	6
	Promotion	1
Function (*n* = 10)	Healthcare professional	1
	Family relatives	2
	Patient with cancer	7
Time from diagnosis until interview in years; median (range) (*n* = 7)		4 [1,3–9,4]
Ongoing treatment (*n* = 7)	Yes	3
	No	4
Time from completed treatment until interview in years; median (range) (*n* = 4)		5,0 [1,6–8,2]

One interview was conducted in person and nine interviews took place digitally via ZOOM. In seven interviews, the PDF version was tested and in three interviews the version on LimeSurvey [50]. The interviews lasted on average 32 min (range: 15–56 min).

### Content analysis

The following nine main categories with 34 subcategories emerged from the predefined category set and the content analysis: ’first impression’, ‘acceptance’, ’completeness’, ‘table of options’, ‘question prompt lists’, ‘user-friendliness/usability’, ’process of handling’, ‘graphical presentation and comprehensibility’ and ‘clarity of the support tool’. IB and LS jointly reviewed the entire text material and achieved an agreement on code selection and length.

#### First impression

At first glance, the participants found the guide well-structured, easy to understand and very comprehensive, so that it could be helpful in the decision-making process:

*“Yes, this is a very comprehensive decision-making guide that really takes many aspects into account. And I found that very good. Especially in the decision-making process, and you actually asked yourself why something like this has not been available for longer” (A0108, pos. 4)*.

One person commented that the guide’s tone felt too academic or mechanical on the first impression, and expressed a preference for a more personal approach.

#### Acceptance & relevance

The participants noted that it has the potential to visualise a complex situation and encourages reflection and due to its guidance character, it can simplify and accelerate processes and decisions. It was also noted that the guide is very detailed and comprehensive and could also be used for other diseases. An important advantage of the guide is that it prioritizes the self-determination of those affected:

*“Yes, supporting other people by simply letting them know that they are in a situation where they are allowed to feel everything; they can feel worried, they can ask questions, right? And they decide what happens to them, so that would have been the most basic thing for me. So, they are not just a… product being processed; they decide on the next steps. […] Since most patients are left alone with the diagnosis and leave the clinic, I think it’s something they can stick to.” (A0102, pos. 125–127)*.

Particularly helpful and useful were the integrated questions from the QPLs, which can be utilized during a patient-physician consultation, for example.

The respondents considered the expression ‘My cancer’ acceptable and personally appropriate, but they acknowledged that some individuals might find this phrase unsettling. Furthermore, this and the use of the guide could be overwhelming due to a lack of clarity. Additionally, the wording is only suitable when the affected individuals complete the guide themselves. It no longer applies if family members take on this task.

Most of the respondents shared the opinion that the title of the guide “Informed decisions - but how? Decision Guide” was appropriate:

*“I think it’s very fitting, actually, what comes next. Because it really is a guide. […] You are guided and it supports you in the decision-making process. […] In this respect it fits”* (A0108, pos. 149–153).

In addition, respondents expressed a desire for a more personalized approach, especially in the introduction of the decision guide.

#### Completeness

Most respondents were not missing any information or topics in the guide. They would not delete any parts of the guide either:

“*So this is really very, very comprehensive. And all possibilities are covered*” (A0103, pos.137).

Only one respondent found the table with the evaluation options of the various options redundant since the physician would usually specify the best and most promising option.

Proposed additions included more detailed and easy-to-understand instructions for filling in and examples for completing the table of options. In addition, the exact naming of support offers, such as addresses of support services for families, rehabilitation services or self-help groups, as well as other contacts and further literature were desired. Specifically, for the LimeSurvey version, a more personal ending and a more specific hint about the summary and print option was requested.

#### Table of options

The table of options was a frequently discussed aspect of the guide. In the table, patients can systematically list their available options, outline each option’s advantages and disadvantages, and evaluate them using a 5-star rating system ranging from ‘unimportant’ to ‘very important. It did not appear self-explanatory and resulted in confusion. Particularly, the first predefined option in the table “No further diagnostics / none of the proposed treatments” was considered non-intuitive, causing challenges in completing the table and using it effectively. Furthermore, it was noted that it appears more logical to place this option further down in the table, as it does not affect all potential users and is perceived as the “last option”.

The evaluation of the options in the table using a star rating system worked technically well. With regard to the selection of the star rating system, respondents had divergent opinions. Some found the star symbol appropriate, while others felt it was not appropriate in the context of cancer. The use of a rating scale with numbers was recommended as an alternative. However the interviewees reported difficulty both in understanding what information to enter into the table and in evaluating the advantages and disadvantages using the star-based rating system from 0 to 5 format.

#### Question prompt lists

The integrated questions from the QPLs were considered important, decision-supportive, comprehensive and relevant. The questions provide prompts for reflection and serve as a preparation as well as assistance for medical consultations. The way of representation and the number of questions is appropriate. The majority of participants reported that no topic was missing. Two participants were missing a question on rehabilitation services and fertility preservation. Interviewees noted that providing additional space for their personal notes under the question sections could further improve usability and contribute to a higher level of support. The possibility to prioritize the questions in the LimeSurvey [50] version was evaluated as positive. This option does not exist in the PDF version. However, respondents noted that prioritization would still be possible.

#### User-friendliness/usability and process of handling

This category focusses primarily on usability, specifically the use in practice, challenges in the application and factors that could facilitate the application. Respondents uniformly indicated that the application of the technical elements in the guide and the manageability in the PDF version and LimeSurvey version were sufficient:

*“But it was manageable, so I think anyone could handle that”* (A0106, pos. 200).

Participants had no clear preference regarding the format. Both versions have their advantages and disadvantages:

“*But that always has the advantage: Of course, I have now made five marks, tomorrow, when I read it again, I might think, “well, I can just change that.” If I have a questionnaire, […] and I have filled something out, I might have to scribbleover it. I won’t send it, I’ll look like an idiot […]”* (A0103, pos. 270–272) or “*I don’t think I would sit down at the doctor’s office with a tablet or something*” (A0104, pos. 270).

Concerns were raised about the usability of the digital version, particularly for older adults and those less comfortable with technology.It was also observed that the guide might be overwhelming and too comprehensive for individuals dealing with cancer:

*“But when you get a pack like that in front of you, I can imagine that there are a lot of people who would say, ’Oh, that’s way too much for me’ "* (A0103, pos. 140).

Interviewees suggested simplyfing the guide by shortening it, e.g. by using bullet points. They recommended an initial summary, with a full version available for those interested.

#### Graphical presentation

The graphical presentation of the guide was described as appropriate and appealing. The font type and size were easy to read and the colour elements were also well chosen:

*“The colour scheme is excellent*, it’s neutral and cohesive throughout. *"* (A0101, pos. 76).

1. Comprehensibility and Clarity of the support tool.

The guide was primarily regarded as highly comprehensible. There were no issues with comprehension when reading or filling it out. Even for non-native speakers, the guide seems to be formulated in an understandable way:

*“As a non-native speaker […] I found it very important that […] erverything was easy to understand and it was. The writing is very clear”* (A0107, pos. 142–146).

In addition, the guide was described as well-structured and logically designed. As an initial step and for orientation, the clarification of the current situation respectively of the decision context proved to be very helpful.

### Expert reviews

Seven reviews from experts for evidence-based health information (*n* = 4) and psycho-oncologists (*n* = 3) were obtained. The experts were part of the TARGET team. Experts mainly suggested formulations. The clinical experts had some reservations about integrating the option of “no further diagnostic” and “none of the proposed treatments” to the table of options. They also criticised that not all patients would have several options. In general, the experts judged the decision guide as a good support tool for patients.

### Revision

Based on the results of the pre-testing, the guide was revised. Most of the changes were spelling, grammar and layout adjustments. Other major changes are listed in Table 2.

**Table 2:** Identified needs for revision and revision

Identified need for revision	Revision
*Table of options*: The table did not seem self-explanatory to the participants and caused confusion.	The filling out instruction of the table has been adapted and the table itself has been supplemented with instructions for completion.
*QPLs*: The participants would prefer more space for their own notes to the questions.	A free text field for notes was added under each question block.
*QPLs*: Questions on rehabilitation services and fertility.	Two questions were added: “Is there a possibility for me to stay in a rehabilitation facility/follow-up treatment? If so, what could they do for me?” “Can my fertility be affected by the treatment?”
*Phrases*: Texts should be formulated more personally.	The text elements have been revised and more personal formulations (e.g. “Dear patients”) have been added.
*Shortening the guide* could improve comprehension	The introduction has been condensed, reducing text, while free text fields have been added to the QPLs, resulting in a more concise and organized question list
*LimeSurvey*: A more personal ending and a more specific hint about the print option was requested.	The end has been revised and a clear reference to the print option has been added.

The participants desired the exact naming of support offers, such as addresses, contact persons and literature. As this is a generic guide, we decided not to include explicit addresses, contact persons or literature. The last part of the guide contains links to the Cancer Information Service (Krebsinformationsdienst) [54] and German Cancer Aid (Deutsche Krebshilfe) [55], where patients can receive disease-specific information.

We stick to our decision to integrate the option of “no further diagnostic” and “none of the proposed treatments” to the table, since both are essential for patients to understand the benefits and harms of each option (e.g., risk differences) and therefore to make an informed decision which aligns with the criteria set by International Patient Decision Aids Standards (IPDAS) and the NICE Guideline for Shared Decision Making [18, 51]. It also encourages the consideration of these as reasonable options, particularly in the case of palliative care situations where best supportive care may be preferable to active treatment. However, as mentioned above, we have adapted the instructions for completing the table to make it clear to patients that this may or may not be a reasonable option, and that it is necessary to compare the different options with none of the proposed interventions to understand the benefits and harms.

## Discussion

We developed and pre-tested a generic decision guide for patients with cancer to support patient participation in decision-making processes. It contains all information that patients valued relevant and useful to make informed choices. The decision guide is a compenent of the complex intervention TARGET which was developedin accordance with the MRC Framework for Development and Evaluation of Complex Interventions [24]. Patients perceived the decision guide as a supportive tool in the decision-making process. The QPLs in particular were perceived as very useful by the target group. Experts from the fields of psycho-oncology and for evidence-based health information also judged the tool as a helpful option for the target group. Overall, the guide was well accepted and seemed to be a good source of support. It also demonstrated the importance of involving the target group and their preferences and experiences.

SDM and decision aids can have positive effects on patients decision-making processes [4, 7, 8]. The Federal Joint Committee (G-BA) recommends that SDM should be transferred to standard care [56]. For a nationwide implementation, effective tools are essential to active patients and to enable them to participate in the decision-making process. Nevertheless, there is a lack of evidence-based decision aids in oncology in Germany and of research findings regarding guidance instruments [18]. For example, there is low certainty evidence showing that guidance instruments significantly improve patient satisfaction with the decision-making process or the choices they make regarding their healthcare. Further evaluation is required to prove their benefits related to facilitate patient participation in decision-making and informed decisions.

Thus, we decided to develop a generic guide to assist patients with cancer asking for and reflecting on important information about their conditions, the treatment options and their advantages and disadvantages. Patients judged our guide as supportive and as potential assistance in a decision-making process.

The results indicate that our guide is a valuable tool that can be utilized in future research to evaluate its effectiveness as a supportive option for patients during the decision-making process in oncology.

Pre-testing our decision guide with patients and subsequently tailoring it based on their feedback can be seen as a strength of our study. In order to enable patients to participate in the decision-making process and for an optimal use of decision aids, there is a need for well-evaluated and scientifically based, target group-oriented tools for decision support. These tools must have high quality and meet the needs of the potential target group. Therefore, it is essential to involve the target group in the development and piloting process, ensuring their perspectives and needs are adequately addressed [22, 57]. Additionally, for implementation, it is helpful to have a trained treatment team willing to include decision aids and knowledgeable about shared decision making [7].

Another strength of our study is the incorporation of questions from the QPLs, which encourages patients to engage actively and informs them about potential inquiries for their doctors [28, 31]. Compared to general information sheets, QPLs were rated as more helpful by patients [47]. Other studies that have developed and tested QPLs for cancer patients also show that patients find QPLs acceptable and useful [40, 44]. These findings were also reflected in our interviews. The number of questions varied from 11 to 110 questions in the included QPLs. A scoping review also found a wide range of 9-191 questions [58], which means that the number of questions we included is in the middle to lower third of the typical number of questions in a QPL. Our integrated questions cover a wide range of topics and were perceived positively and comprehensively by the study participants. The breadth of topics covered by these questions also enhances the guide’s generic applicability. There is also the added value of having integrated the questions into the instructions for the decision-making process, creating a kind of pre-structuring. It is important that QPLs are tailored, taking into account different attitudes to the disease and to prognosis and involvement in decision making [59]. Future research should explore how effectively patients can utilize these questions during consultations, particularly given the constraints of limited appointment time. It would be further desirable to integrate the decision guide in a patient app in the future which would allow a high dissemination potential and ensure practical use at the same time. However, it has to be considered that our developed decision guide will be part of a structured counselling process provided by trained health professionals. This should be taken into consideration for future research or implementation strategies. Nevertheless, other decision guidance instruments are also available for the use of patients alone.

Our study has some limitations. It is possible that relevant QPL studies were not included because only German and English studies could be considered. The patients involved in the pre-testing process could only imagine using the guide in decision-making or recall how it might have influenced their past decisions. Consequently, the results may be biased due to e.g. emotional influences on their memories. However, participants shared insights from their experiences, highlighting what they needed for decision-making at the time, what was lacking, and what went wrong. The planned process evaluation of the whole project will examine whether the decision guide is effective in supporting real decision-making processes.In addition, a selection bias cannot be excluded, as we were only partially successful in achieving a well-balanced sample. We were unable to include young cancer patients, and the representation of individuals with high and low levels of education is imbalanced. The absence of younger patients, means that the experiences, needs and perspectives of this group may not be represented. Younger patients may have distinct priorities that could influence the development and use of the decision guide. Additionally, the imbalance in education levels may have biased the results in terms of comprehension, limiting generalizability across all cancer patients. These factors should be considered when interpreting the findings. Further research involving a more diverse group of participants is needed. Therefore, the LimeSurvey [50] version could only be tested with three participants. However, due to the same content as the PDF version, this is not seen as a major limitation.

## Conclusions

Our interview study indicates that participants view the decision guide as a and well-accepted support tool for cancer patients facing medical decisions related to their disease. In line with this, the guide appears to be useful, comprehensible, attractive, and user-friendly for the target group based on the preliminary evidence presented; however, further research is needed to confirm these findings. It will be crucial to evaluate the guide with patients of different ages and those with lower educational levels, as well as with patients who have actively used the guide in a decision-making situation. Further steps are needed to integrate the guide into care structures, necessitating the development and testing of effective implementation strategies. Within the TARGET project, it is being made available to patients and can be combined with a decision coaching. A process evaluation is also planned as part of the TARGET project. Exploring digital options to enhance accessibility for patients should also be considered.

## Electronic supplementary material

Below is the link to the electronic supplementary material.

Supplementary Material 1

Supplementary Material 2

Supplementary Material 3

Supplementary Material 4

Supplementary Material 5

Supplementary Material 6

## Data Availability

Full access to the raw data is available upon reasonable request.

## Abbreviations

IPDAS: International Patient Decision Aid Standards
MRC: Medical Research Council
OPDG: Ottawa Personal Decision Guide
ODSF: Ottawa Decision Support Framework
QPL: Question Prompt List
SDM: Shared Decision Making
