# Supplementary material for: Development of a generic decision guide for patients in oncology: a qualitative interview study
Source: BMC Med Inform Decis Mak. 2025 Mar 10;25:125. doi: 10.1186/s12911-025-02960-6 (PMC11895154; doi:10.1186/s12911-025-02960-6)
Supplement: Supplementary file 1 — Supplementary Material 1 [file 12911_2025_2960_MOESM1_ESM.docx]

Additional file 1: CReDECI 2 checklist

| **Item** | **Reported on page or in publication** |
| --- | --- |
| **First stage: Development**   1. Description of the intervention’s underlying theoretical basis 2. Description of all intervention components, including the reasons for their selection as well as their aims / essential functions 3. Illustration of any intended interactions between different components 4. Description and consideration of the context’s characteristics in intervention modelling   **Second stage: Feasibility and piloting**   1. Description of the pilot test and its impact on the definite intervention   **Third stage: Evaluation**   1. Description of the control condition (comparator) and reasons for the selection 2. Description of the strategy for delivering the intervention within the study context 3. Description of all materials or tools used delivery the intervention 4. Description of fidelity of the delivery process compared the study protocol 5. Description of a process evaluation and its underlying theoretical basis 6. Description of internal facilitators and barriers potentially influencing the delivery of the intervention as revealed by the process evaluation 7. Description of external conditions or factors occurring during the study which might have influenced the delivery of the intervention or mode of action (how it works) 8. Description of costs or required resources for the delivery of the intervention | 6 f  6 f  6 f  6 f  6, 8 ff  n.a.  n.a.  n.a.  n.a.  n.a.  n.a.  n.a.  n.a. |
