# Supplementary material for: Development of a generic decision guide for patients in oncology: a qualitative interview study
Source: BMC Med Inform Decis Mak. 2025 Mar 10;25:125. doi: 10.1186/s12911-025-02960-6 (PMC11895154; doi:10.1186/s12911-025-02960-6)
Supplement: Supplementary file 3 — Supplementary Material 3 [file 12911_2025_2960_MOESM3_ESM.docx]

Additional file 3: search string for Medline via PubMed, from 15.08.2022

| ("oncolog*"[All Fields] OR "cancer*"[All Fields] OR "tumor*"[All Fields] OR "neoplas*"[All Fields] OR "malignan*"[All Fields] OR "neoplasms"[MeSH Terms])  **AND**  ("question prompt sheet"[All Fields] OR "question prompt"[All Fields] OR "question prompts"[All Fields] OR "question prompt list"[All Fields] OR "patient questions"[All Fields] OR "patient question lists"[All Fields] OR "patient question prompt"[All Fields] OR "asking questions"[All Fields] OR "question asking"[All Fields] OR "patient question asking"[All Fields] OR "prompt list"[All Fields] OR "prompt sheet"[All Fields] OR "prompt tool"[All Fields]) |
| --- |
