# Supplementary material for: Development of a generic decision guide for patients in oncology: a qualitative interview study
Source: BMC Med Inform Decis Mak. 2025 Mar 10;25:125. doi: 10.1186/s12911-025-02960-6 (PMC11895154; doi:10.1186/s12911-025-02960-6)
Supplement: Supplementary file 4 — Supplementary Material 4 [file 12911_2025_2960_MOESM4_ESM.docx]

**Additional file 4: Material Testing Interview Guide - Decision Guide: PDF or Paper-Testing for Clarity, Acceptance, Completeness**

| **Interview** | | |
| --- | --- | --- |
| **Introduction**  This study is a project within the study TARGET. We are focused on supporting informed decision-making for people with (rare) cancers and their relatives. As part of our study, we will discuss the decision guide. | | |
|  | | |
| **Questions/subject** | **Follow-up questions (for elaboration/probing)** | Notes |
| Did you have the opportunity to read and possibly edit the decision guide beforehand? | - Did you read/edit all of it or only parts? - If only parts: Which parts? - Did you work through the decision guide from the beginning to the end or did you "jump" between topics/sections? |  |
| Can you describe your initial impression of this decision guide? | Can you explain in your own words what the decision guide is for? |  |
| **User friendliness/Usability** |  |  |
| How did you find entering information? | - Did you edit the decision guide on the computer or did you use a printed copy? - Did you know what to enter in each field, or were there uncertainties?   Follow-up questions specifically about certain pages:   - Page 7: How did you find the star rating? - If using digital PDF: How did you find the font size in the fillable fields? |  |
| Is there anything that would make the use of the tool easier for you? | If yes, what would that be? |  |
| Why did you choose not to edit/open certain sections? |  |  |
| How did you find the usability of the decision guide? |  |  |
| **Comprehensibility und clarity** | |  |
| Which decision situation did you choose? | In which category would you classify this decision situation on page 4 (From your perspective, is it more diagnostic or therapeutic)? |  |
| How do you find the structure of the decision guide? | - How well did you navigate through it? (Order of chapters/question categories.) - Where did you continue after page 6 (did you know where to proceed next in the decision guide)? |  |
| How did you find the collection of questions in the decision guide? | - How did you perceive the number of questions? - Was the presentation in tabular form clear and understandable? - Did you know how to fill out the table of questions? - How many questions did you select/check? - How did you find the order of questions within the tables? - Were there any questions missing and that would have been of interest to you? - If yes, would these questions also be relevant for decision-making? - Do you consider any questions less important? - Which questions would you choose and in what hierarchy? How many questions are realistically manageable? - Based on the questions you selected, would you be able to prioritize them for yourself in a doctor's appointment? What else would you need for that? Should there be guidance on prioritizing the selected questions? |  |
| What was unclear or difficult for you to understand? | Where did problems/difficulties arise in the application? |  |
| **Completeness** | |  |
| Did you miss anything in the decision guide that you need in order to decide? | Which one is that? |  |
|  | What specific information are you lacking regarding supplementary details? |  |
| If you could express preferences, which parts of the decision guide would you: | - remove - definitely keep |  |
| **Acceptance** | |  |
| When reflecting on your questions about diagnostic and/or treatment decisions, how would this decision guide have supported you in making those decisions? | What need for additional or differently presented information did you have? |  |
| How can you imagine that this guide supports individuals in their decision-making process? | What do you feel might be missing from your perspective? |  |
| How did you perceive the wordings used? | e.g. my cancer |  |
| Is the title "Decision Guide" appropriate, or how would you name it? |  |  |
| No treatment/diagnosis | How did you feel about the placement of "No treatment/diagnosis"?  Page 7, table with the options |  |
| **Graphical representation** | |  |
| How do you find the graphical presentation of the decision guide?  Clarity of the decision guide? | - Color differentiation - Font type - Font size - Page 13, gray box: readability? - Page 14, green text: readability, link recognized + functioning? - Symbols used, do you like them? - Table with pros and cons | if not already described at the beginning |
| Thank you very much for your participation and feedback. Is there anything else you would like to share with us that you feel hasn't been covered yet? |  |  |
