# Supplementary material for: Development of a generic decision guide for patients in oncology: a qualitative interview study
Source: BMC Med Inform Decis Mak. 2025 Mar 10;25:125. doi: 10.1186/s12911-025-02960-6 (PMC11895154; doi:10.1186/s12911-025-02960-6)
Supplement: Supplementary file 5 — Supplementary Material 5 [file 12911_2025_2960_MOESM5_ESM.docx]

Additional file 5: category system

| **Main category** | **Subcategories** | **Examples for the main category’s** |
| --- | --- | --- |
| Process of handling | Edited sections  Order of reading | "I read it completely, I even read it twice, very carefully." (A01018, pos. 6-8) |
| First Impression | Positive  Negative | "Yes, this is a very detailed decision-making guide that really takes many aspects into consideration. I thought that was very good, especially in the decision-making process and I actually asked myself why something like this hasn't been around longer." (A0108, pos. 4) |
| User-friendliness/usability | Medium  Paper  Digital  Simplification of the application  Highlighting  Larger fields  Simplify language  Changing the order  Reduction  Usability  Use in practice  Good (technical) usability  Problems  External links  LimeSurvey technical application  Drag and drop function / order  Thumb function  Print version  Saving function | "I mean, I'm a bit older now. But I'm still quite good with electronic things. It was easy to handle, so I think anyone could do it." (A0106, pos. 196-200) |
| Clarity of the support tool | Good comprehensibility  Structure of the guide | "It was plausible […] I think that's logical." (A0101, pos. 32) |
| Completeness | Length of the guide  Unnecessary components  Suggested additions/missing information  Text elements  Reference to support services  Emotional aspects  No missing information | “What I was missing a bit was where there are self-help groups." (A0106, pos. 381) |
| Acceptance | Decision situation  Formulations used  More personal formulations  Spelling/grammar  Terms used  Title of the guide  “My cancer”  Usefulness/degree of support of the guide | "...if the decision guide is in the hands of the patient at some point, that's a good thing. I think." (A0105, pos. 34) |
| Graphical presentation and comprehensibility | Overall impression  Typeface  Color differentiation  Other | "I thought that was very good. So it wasn’t over the top, colorful in any way, but very coherent in the overall picture of the guide. So I liked that a lot." (A0108, pos 137-138) |
| Table of options | Placement “no treatment”/“no diagnostics”  Usability  Usefulness  Question mark function  Incomprehensibility  Star function | "For example, on page 7, where the stars are, I have no idea what to do with them." (A0102, pos. 22) |
| Question Prompt Lists | Prioritization  Order  Relevance  Presentation  Usability  Number of questions  Missing questions  No missing questions | "in one situation or another, I might think of an additional question, but I found that the questions listed in the guide were very comprehensive and covered many areas." (A01015, pos. 69-71) |
