# Supplementary material for: Development of a generic decision guide for patients in oncology: a qualitative interview study
Source: BMC Med Inform Decis Mak. 2025 Mar 10;25:125. doi: 10.1186/s12911-025-02960-6 (PMC11895154; doi:10.1186/s12911-025-02960-6)
Supplement: Supplementary file 6 — Supplementary Material 6 [file 12911_2025_2960_MOESM6_ESM.pdf]

University of Bremen

TARGET
Vernetzte Versorgung bei Krebs

Informed decision-making - but how?

Decision guide

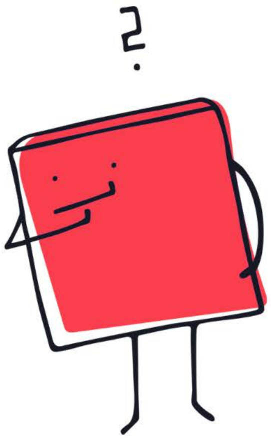

What you should know about using the decision guide!

Dear patients,

this decision guide has been developed for individuals facing a decision about how to deal with their cancer diagnosis. You can use this guide as a support to make personal decisions, for example about possible treatments or tests.

Today, many people want to be involved in decisions concerning their health. Often, there are several options for further action, each with different advantages and disadvantages. In order to make the right decision for yourseld, it is important to be aware of these pros and cons.

Your test results are often discussed by a team of experts - also known as a tumor board. A recommendation is made based on current research and medical findings. Your healthcare team will then discuss the options with you.

TARGET
Vernetzte Versorgung bei Krebs

In order to make a decision based on your personal preferences and individul circumstances, it is important that you ask any questions and let your medical team know what is important to you personally and what concerns you have.

The decision guide helps you to make structured notes so that you and your healthcare team can make a decision about how to proceed. It aims to assist you in understanding where you stand in your treatment journey and what options are available to manage your situation. It summarizes your current status and does not provide separate patient information.

If you wish, you can bring your decision guide to further discussions with your healthcare team so that your open points can be addressed. If you have further questions, please feel free to reach out to your healthcare team or your oncology coach.

The decision guide is divided into various steps

1. My Question
2. My Options
3. What am I still missing?

1. My Question

What decision do you face?

What are your reasons for making this decision?

When do you neet to make a choice?

What type of decision is it?

☐ It is a decision regarding a diagnostic test.

☐ It is a decision regarding a treatment.

☐ It is another decision, specifically about: ____________________.

How far along are you with making a choice?

☐ Not thought about it.

☐ Thinking about it.

☐ Close to choosing.

☐ Made a choice.

Where do I stand?

At this point, you can take notes on your findings and diagnosis. If you lack information, feel free to speak to your treating oncologist.

What diagnostic tests have been conducted so far?

Where is my cancer?

What do the results of the tests so far mean?

At what stage is my cancer?

What is the prognosis for me if I do not undergo treatment?

What symptoms does the cancer cause?

Additional notes on your findings:

2. My Options

There are usually various treatment or diagnostic options available. This may include doing nothing or no further treatment, as an equally valid option.

Once you have all the necessary information, you can compare the different diagnostic and treatment options and weigh up their potential benefits and harms. It is important that you know your needs and preferences and communicate them to your healthcare team.

The following pages are intended to help you to become aware of your personal needs and preferences regarding your next steps. To weight up the benefits and harms of a proposed treatment, it is important to know what outcomes you can expect without treatment or the proposed diagnostics.
You can use the table on the next page for this purpose.

- Check the box next to Option 1 to indicate which alternative you want to compare to the proposed treatment/diagnostic.
- List your different options.
- Take a look at them and add all the important pros an cons for each option for yourself.
- Use stars to indicate how important each pro and con is to you. 5 stars signify "very important," and no star means it's "unimportant" to you.

To receive guidance on filling out the form, please click on the question mark symbols ? in the table

Option 1: (please check the appropriate box) ?

☐ no further diagnostic ☐ none of the proposed treatments ☐ none ______________________

Benefits / Advantages / Pro ?	How much it matters to you?* ?	Risks / Disadvantages / Cons ?	How much it matters to you?* ?
	☆☆☆☆☆		☆☆☆☆☆
	☆☆☆☆☆		☆☆☆☆☆
	☆☆☆☆☆		☆☆☆☆☆
	☆☆☆☆☆		☆☆☆☆☆

*0 stars = not at all to 5 stars = a great deal

Option 2:

____

Benefits / Advantages / Pro	How much it matters to you?*	Risks / Disadvantages / Cons	How much it matters to you?*
	☆☆☆☆☆		☆☆☆☆☆
	☆☆☆☆☆		☆☆☆☆☆
	☆☆☆☆☆		☆☆☆☆☆
	☆☆☆☆☆		☆☆☆☆☆

*0 stars = not at all to 5 stars = a great deal

Option 3:

____

Benefits / Advantages / Pro	How much it matters to you?*	Risks / Disadvantages / Cons	How much it matters to you?*
	☆☆☆☆☆		☆☆☆☆☆
	☆☆☆☆☆		☆☆☆☆☆
	☆☆☆☆☆		☆☆☆☆☆
	☆☆☆☆☆		☆☆☆☆☆

*0 stars = not at all to 5 stars = a great deal

Your results are often discussed by experts in a tumor board and a recommendation for further action is made. If you know these, you can make a note of the tumor board's recommendation here.

The recommendation of the tumor board is:

____

What other questions do I have?

You may still lack important information to make a decision. We have compiled a list of possible questions that you can discuss with your treatment team.
Please check the questions you would like to discuss with your treatment team and consider which checked questions are particularly important to you in preparation for your doctor's appointment. If you have additional questions, you can note them below the question blocks

Questions about diagnostic examinations

- ☐ Which further diagnostic examinations are suitable for me?
- ☐ What is the purpose/objective of the diagnostic examination?
- ☐ How reliable are the examination results?
- ☐ What is involved in the diagnostic procedure: at what times, how often, for how long each time, and over what total period, where will the examination take place?
- ☐ How likely is it that the examination will reveal further treatment options or improve the predictability of the success of certain treatments?
- ☐ What risks and side effects are associated with the examination?

My additional questions:

Questions about prognosis

- ☐ Is it possible to cure my cancer?
- ☐ What happens if my cancer is left untreated?
- ☐ What symptoms will it cause if left untreated?
- ☐ What is the life expectancy for people with my cancer diagnosis typically without treatment?
- ☐ Can my tumor regress on its own?
- ☐ How likely is it that my cancer will spread to other areas of my body?
- ☐ How likely is it that the treatment will control my cancer?
- ☐ If the cancer can be cured, what is the likelihood of it recurring?

My additional questions:

Questions about treatment options

- ☐ What is the goal of the proposed treatment? (Shrink the tumor / make it operable / prevent growth / maintain the status quo)
- ☐ What are the other treatment options available?
- ☐ What are the pros and cons of each treatment option?
- ☐ What is the best-case scenario that could result from the treatment?
- ☐ What is the most likely outcome of the treatment?
- ☐ How likely is it that my cancer will spread to other areas of my body?
- ☐ How likely is it that I will be cured by each treatment option?

- ☐ If my cancer can no longer be cured, how much additional time can I gain from treatment?
- ☐ What type of side effects are there? How likely are these side effects?
- ☐ What is involved in the treatment: at what times, how often, for how long each time, and over what total period, where will the treatment take place?
- ☐ Which treatment is recommended by you or by the tumor board?
- ☐ How much time do I have to make a decision?
- ☐ Can I wait to start treatment? What are the pros and cons of waiting? How long can I wait?

My additional questions:

Questions about symptom and side effect management

- ☐ What symptoms may arise from the treatment or the cancer in the future, and what options are available to alleviate them (such as pain, discomfort, constipation, shortness of breath, nausea or vomiting, loss of appetite, fatigue, dry mouth)?
- ☐ What alternative healing methods are available to reduce side effects or symptoms?
- ☐ What can I actively do to avoid side effects? How can I take good care of myself beyond that?
- ☐ What assistance will I need in coping with the side effects? When and for how long?

- ☐ Do the side effects improve or worsen as the treatment progresses? How long will they last?
- ☐ Does the treatment affect other health issues (such as diabetes, hypertension)?
- ☐ Can my fertility be affected by the treatment?

My additional questions:

Questions about participating in clinical trials

- ☐ Where can I get information about currently ongoing studies?
- ☐ Are there any studies I can participate in?
- ☐ For which clinical trials am I eligible? What makes me eligible and what doesn't?
- ☐ What is the goal of the clinical trial?
- ☐ What benefits will I gain from participating in the study?
- ☐ What risks and side effects are associated with the new treatment? Are there any permanent or long-term side effects? Are there any serious or rare side effects I should be aware of?
- ☐ Has the benefit of the new treatment already been proven in people like me?
- ☐ What is already known about the effectiveness of the new treatment compared to standard treatment?
- ☐ What is involved in the clinical trial: at what times, how often, for how long each time, over what total period, and at what location will the treatment be administered?
- ☐ How much time do I have to decide whether to participate in the study? Does the time I need to make the decision affect the effectiveness of the treatment?

- ☐ If I participate in the study and later change my mind, how can I withdraw from participation?
- ☐ If I participate in the clinical trial, will the pharmaceutical company cover my treatment costs, or will they be covered by health insurance?
- ☐ Do I know which treatment I will receive, or is the study blinded? What does that mean and why is it important for the study? Will I ever find out what I received?
- ☐ Can I still use alternative healing methods if I participate in the study (e.g., vitamins, herbal remedies, naturopathy, dietary changes)?
- ☐ If the new treatment proves to be effective, will I still have access to it after the study ends?
- ☐ If the new treatment proves to be ineffective, will I have access to standard treatment? Will it still be possible, and will there be any disadvantages to me from participating in the study?

My additional questions:

Questions about support

- ☐ What support options are available to me?
- ☐ Are there organizations or services I can contact (e.g., for respite care, disabled person's pass)?
- ☐ Is there a possibility for me to stay in a rehabilitation facility/follow-up treatment? If so, what could they do for me?
- ☐ Who can I talk to about my spiritual, religious, and emotional needs?
- ☐ What support services are available now and in the future for my family and children?

My additional questions:

Questions about palliative care

- ☐ What is palliative care and what does it encompass? Do you believe it could help me?
- ☐ What types of palliative care are available, and which type would be suitable for me?
- ☐ When would it be beneficial for me to seek palliative care?
- ☐ Who provides this care?
- ☐ How/where can I get information about palliative care?
- ☐ What can I expect in the last days of my life?
- ☐ Who can I talk to now about my advance directives, power of attorney, and the medical care I want in the future if I am unable to speak for myself?
- ☐ Are there organizations or services I can contact for support (e.g., hospice service)?

My additional questions:

3. What am I missing?

Support

Who else is involved?

Which option do they prefer?

How can they support you?

Is a person pressuring you? If so, who?

☐ yes ______________________________

☐ no

What role do you prefer in making this choice?

☐ Share this decision with ______________ .

☐ Decide myself after hearing views of

______________________________.

☐ Someone else decides

____.

Identify your decision making needs

**Knowledge**	Do you know the benefits and risks of each option?	☐ yes<br>☐ no
**Values**	Are you clear about which benefits and risks matter most to you?	☐ yes<br>☐ no
**Support**	Do you have enough support and advice to make a choice?	☐ yes<br>☐ no
**Certainty**	Do you feel sure about the best choice for you?	☐ yes<br>☐ no

People who answer "No" to one or more of these questions are more likely to delay their decision, change their mind, feel regret about their choice or blame others for bad outcome. It is therefore important to go through the following steps, which focus on your needs. If you would like further support, there is the option to seek decision coaching.

What else do you need for the decision?
What could help you with the preparation?

Knowledge

If you feel you do NOT have enough facts

- ☐ Find out more about the options (p.6) and the chances of the benefits and risks.
- ☐ Bring your collected questions (from p. 7 onwards) to the doctor's appointment.
- ☐ Look for trustworthy information (e.g. cancer information service, German Cancer Aid, onco-coach)

Values

If you are NOT sure which benefits and risks matter most to you

- ☐ Review the stars (p. 6) to see what matters most to you.
- ☐ Find people who know what it is like to experience the benefits and risks.
- ☐ Talk to others who have made the decision.
- ☐ Read stories of what mattered most to others.
- ☐ Discuss with others what matters most to you. (e.g. relatives, friends, psycho-oncologists).

Support

If you feel you do NOT have enough support:

- ☐ Discuss your options with a trusted person (e.g. health professional, Onco-coach, counsellor, family, friends).
- ☐ Find help to support your choice (e.g. funds, transport, child care).
- ☐ Tell your psycho-oncologist or relatives and friends that you need support. If possible, specify exactly what support you need (e.g. financial or psychosocial).

If you feel PRESSURE from others to make a specific choice:

- ☐ Focus on the views of others who matter most.
- ☐ Share your decision guide with others.
- ☐ Ask others to fill in this decision guide. (See where you agree. If you disagree on facts, get more information. If you disagree on what matters most, consider the other person's views. Take turns to listen to what the other person says matters most to them.)
- ☐ Find a person to help you and others involved (e.g. in the form of professional support).

Other factors making the decision DIFFICULT:

Please write down here what else you need for your decision:

What can you do next:

Once you've made a decision, the next step is to proceed with its implementation. You can discuss with your healthcare team how the decision will be carried out and come to an agreement. Feel free to document these agreements here.

I have made the following agreements with my treatment team regarding the implementation of my decision:

To review your decision at a later time and possibly make changes, **you can note here whether and when you would like to revisit your decision:**

References

Ottawa Personal Decision Guide. O‘Connor, Stacey, Jacobsen 2012. Ottawa Hospital Research Institute and University of Ottawa, Canada. [German translation and adaption: IQWiG 2013]

Imprint

Publisher: University of Bremen, Institute for Public Health and Nursing Research, Department of Nursing Science Evaluation and Implementation Research, Grazer Str. 4, 28359 Bremen
Responsible: Prof. Dr. Birte Berger-Höger, Department of Nursing Science Evaluation and Implementation Research
Date: 07.03.2023
Planned Update: 01.01.2025

Who developed the decision guide?

This decision guide is based on the Ottawa Personal Decision Guide (O'Connor, Stacey, Jacobsen 2015) and was adapted to the special needs of cancer patients in order to support informed choices and shared decision-making. It is part of a trans-sectoral personalized care concept for patients with rare and advanced cancer (TARGET) and is used by those affected to decide on the best course of action in managing their cancer.

It has been tested with affected individuals for completeness and comprehensibility.

The decision guide was created by Lia Schilling, Jana Kaden, and Prof. Dr. Birte Berger-Höger. We thank Dr. med. Friederike Mumm, Theresia Pichler, and Dr. med. C. Benedikt Westphalen from CCC München - Comprehensive Cancer Center, as well as Prof. Dr. Anke Steckelberg, Dr. Julia Lühnen, Jana Hinneburg, and Sandro Zacher from Martin-Luther-Universität Halle-Wittenberg for their content support, and Ann-Marie Pick for assistance with implementation.
This project is funded by the Innovation Fund of the Joint Federal Committee (Funding Code 01NVF20012).
